# New Environment-Sensitive Multichannel DNA Fluorescent Label for Investigation of the Protein-DNA Interactions

**DOI:** 10.1371/journal.pone.0100007

**Published:** 2014-06-12

**Authors:** Alexandra A. Kuznetsova, Nikita A. Kuznetsov, Yuri N. Vorobjev, Nicolas P. F. Barthes, Benoît Y. Michel, Alain Burger, Olga S. Fedorova

**Affiliations:** 1 Siberian Branch of the Russian Academy of Sciences, Institute of Chemical Biology and Fundamental Medicine, Novosibirsk, Russia and Department of Natural Sciences, Novosibirsk State University, Novosibirsk, Russia; 2 Institut de Chimie de Nice, UMR 7272, Université de Nice Sophia Antipolis, CNRS, Nice, France; University of South Florida College of Medicine, United States of America

## Abstract

Here, we report the study of a new multichannel DNA fluorescent base analogue 3-hydroxychromone (3HC) to evaluate its suitability as a fluorescent reporter probe of structural transitions during protein-DNA interactions and its comparison with the current commercially available 2-aminopurine (aPu), pyrrolocytosine (C^py^) and 1,3-diaza-2-oxophenoxazine (tC^O^). For this purpose, fluorescent base analogues were incorporated into DNA helix on the opposite or on the 5′-side of the damaged nucleoside 5,6-dihydrouridine (DHU), which is specifically recognized and removed by Endonuclease VIII. These fluorophores demonstrated different sensitivities to the DNA helix conformational changes. The highest sensitivity and the most detailed information about the conformational changes of DNA induced by protein binding and processing were obtained using the 3HC probe. The application of this new artificial fluorescent DNA base is a very useful tool for the studies of complex mechanisms of protein-DNA interactions. Using 3HC biosensor, the kinetic mechanism of Endonuclease VIII action was specified.

## Introduction

**Figure 1:**
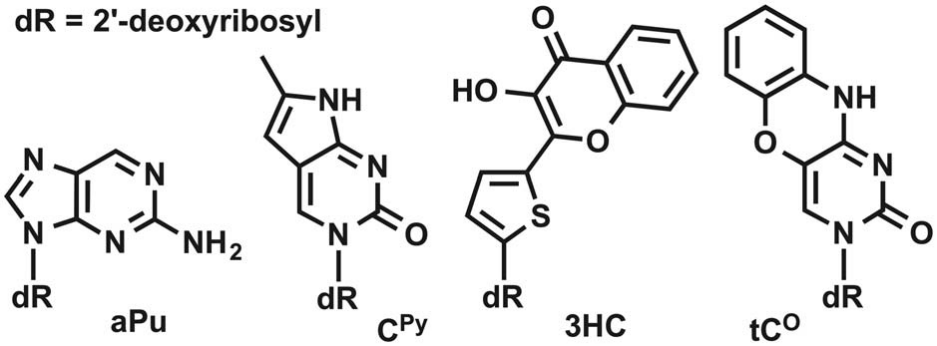
Structures of the fluorescent base analogues.

Understanding at the molecular level the dynamics and functions of enzymes in interactions with their DNA targets is of main importance in biology and medicine. In this context, fluorescence spectroscopy is widely used in nucleic acids research to study the structure and the dynamics as well as the kinetics of protein-DNA interactions [1]. Different fluorescent analogues of DNA bases have been evaluated for examining of DNA conformational transitions [2]–[8]. Most of them are sensitive to the quenching of DNA nucleobases. The degree of quenching depends on the conformation of DNA and on the composition of the bases surrounding the fluorescent dye. The sensitivity to nucleobase quenching has therefore been exploited to study in real time the dynamic and function of nucleic acids. The most commonly used fluorescent base analogue is 2-aminopurine (aPu, Fig. 1). 2-Aminopurine can form stable base pairs with thymine but also moderately stable base pairs with cytosine [9]. The sensitivity of aPu fluorescence to the microenvironment has been utilized in the study of wide range protein-DNA interactions [10]–[14]. The cytosine analogue pyrrolocytosine (3-[*β*-D-2-ribofuranosyl]-6-methylpyrrolo[2,3-d]pyrimidin-2(3H)-one, C^py^, Fig. 1) forms hydrogen bonds with guanine. In some studies, the DNA duplexes containing C^py^ as a fluorescent reporter were used for the investigation of their bindings with some proteins [15]–[17]. Moreover, the tricyclic cytosine analogue as 1,3-diaza-2-oxophenoxazine (tC^O^, Fig. 1) was reported as a fluorescent cytosine analogue and recently proposed for studying the physical properties of DNA and protein-DNA interactions [18]–[21].

**Figure 2:**
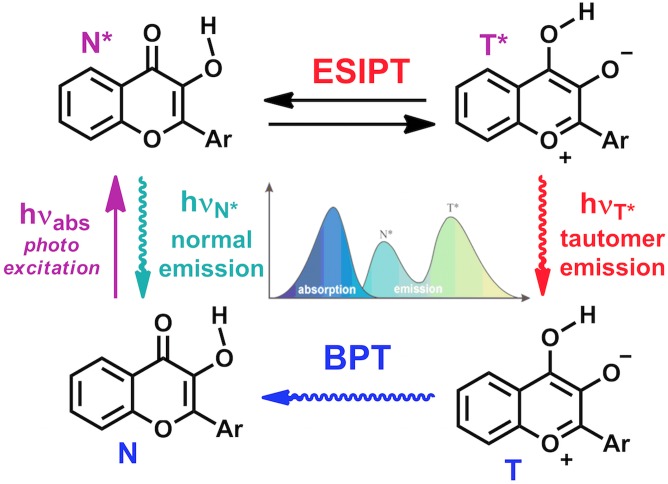
Origin of the dual emission of 3-hydroxychromones: the ESIPT reaction. BPT denotes back proton transfer; N* and T* represent the normal and tautomeric emissive forms, respectively.

The design of new DNA-based fluorescent labels, with high site-specific responses to intermolecular interactions and sensitive to the environmental change by a different mechanism than quenching, attracts great attention of chemists. New probes are desirable to overcome the limited environment sensitivity of the dye when strongly quenched and to get further insight on the molecular mechanisms of their interactions by sensing new mechanisms of the fluorescent signal response. Recently, the interesting fluorescent properties of the base analogue 3-hydroxychromone (3HC, Fig. 1) have been reported [22], [23]. This probe is up to 50-fold brighter than 2-aminopurine and can substitute any nucleobase in a duplex with minimal perturbation of the duplex structure. The total emission of 3HCs is sensitive to G, C and water mediated quenching, but not A and T. In addition, due to an excited state intramolecular proton transfer (ESIPT), 3HC fluorophores exhibit two excited states: the initially excited normal form (N*) and the tautomeric (T*) one; each form generates one well-resolved emission band (Fig. 2) [24]. The dual emission of 3HCs is highly sensitive to the polarity of the environment because an increase in the hydrogen bond donor strength and the dielectric constant of solvents inhibit the ESIPT reaction and thus, decrease the relative intensity of the T* band [23], [25], [26]. Therefore the variation of dipole/dipole interactions and hydration of the microenvironment of 3HC can be monitored by measuring the intensity ratio of the two emission bands (I_N*_/I_T*_). Taken together, 3HC labeled oligodeoxynucleotides (ODNs) provide different channels of information: the intensiometric channel based on sensing quenching and the ratiometric channel based on sensing hydrogen bond donor strength and dipole/dipole interactions of 3HC microenvironment. An important advantage of the ratiometric dye over conventional single-band intensiometric dyes is that this ratio is independent from unaccountable quenching effects or instrument settings. In addition, 3HC based sensors distinguish from the majority of ratiometric sensors, which employed fluorescence resonance energy transfer (FRET) pairs or excimers and required double labeling. Therefore, 3HC labeled ODNs are prospective sensors for the studies of interactions with proteins [23].

**Figure 3:**
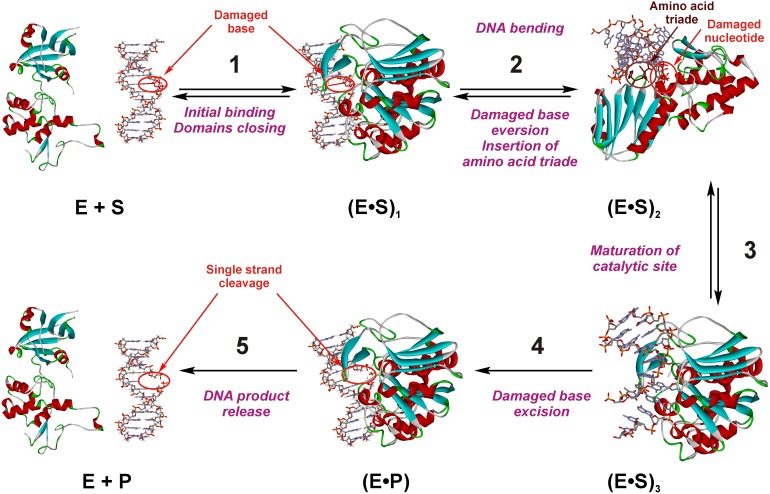
The proposed kinetic mechanism of Nei processing on the DNA substrate containing DHU [34]. Crystal structures of Nei (PDB ID 1Q39 and 1K3W) were used for schematic representation of the enzyme•DNA complexes.

**Figure 4:**
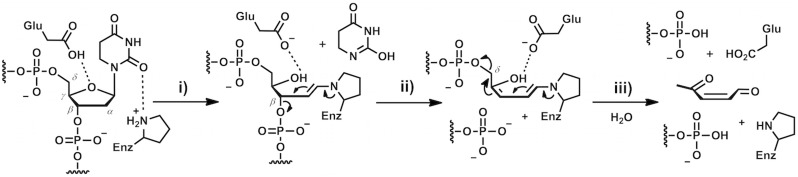
Main chemical steps of Nei catalysis. Step i: acid activation of the nucleobase and Schiff base formation; step ii: *β*-elimination of 3′-phosphate, step iii: *δ*-elimination of 5′-phosphate.

The first objective of this work is a comparative study of the new 3HC fluorescent nucleobase moiety with current commercially available dyes (aPu, C^py^, tC^O^) according to the following questions. Is the non-natural dye a suitable fluorescent base substitute for monitoring complex mechanism of DNA structural transitions during interaction with a protein [27]? Can the multi-channel probe complement the information obtained from conventional dyes? To address these questions, the Endonuclease VIII from *Escherichia coli* (Nei) was used as a model. Nei is a DNA repair enzyme that removes a wide range of damaged pyrimidine bases from DNA such as 5,6-dihydrouracil (DHU), thymine glycol, 5,6-dihydrothymine, 5-hydroxypyrimidines, etc [28], [29]. Based on crystal structures of the enzyme free and in complex with the target DNA [30], [31], biochemical means [32], [33] and stopped flow studies of the protein dynamics [34], a mechanism was proposed to explain how Nei process on the DNA substrate. The recognition of DNA lesions by Nei involves several conformational changes in both protein and DNA, such as DNA kinking, damaged base flipping out from DNA helix, insertion into the enzyme’s active site and the intrusion of the enzyme loop (a triad: Gln-69, Leu-70, and Tyr-71) into the void created in DNA after eversion of the damaged base, damaged base excision and product release (Fig. 3). It was shown [29] that the excision of the damaged base by Nei proceeds through several chemical steps: *N*-glycosidic bond cleavage (Fig. 4, stage i), *β*-elimination (Fig. 4, stage ii) and *δ*-elimination (Fig. 4, stage iii) of the phosphates flanking the damaged nucleoside. Since the mechanism involves several conformational changes in both protein and DNA, monitoring the DNA dynamics in time should give further insight about the processing. However, preliminary studies using stopped flow kinetics and aPu labeled DNA with Nei failed to give such information because no significant fluorescence changes of aPu were measured [34]. Therefore, the second objective of this work is to fill this gap and complement the information collected following the protein dynamics.

**Figure 5:**
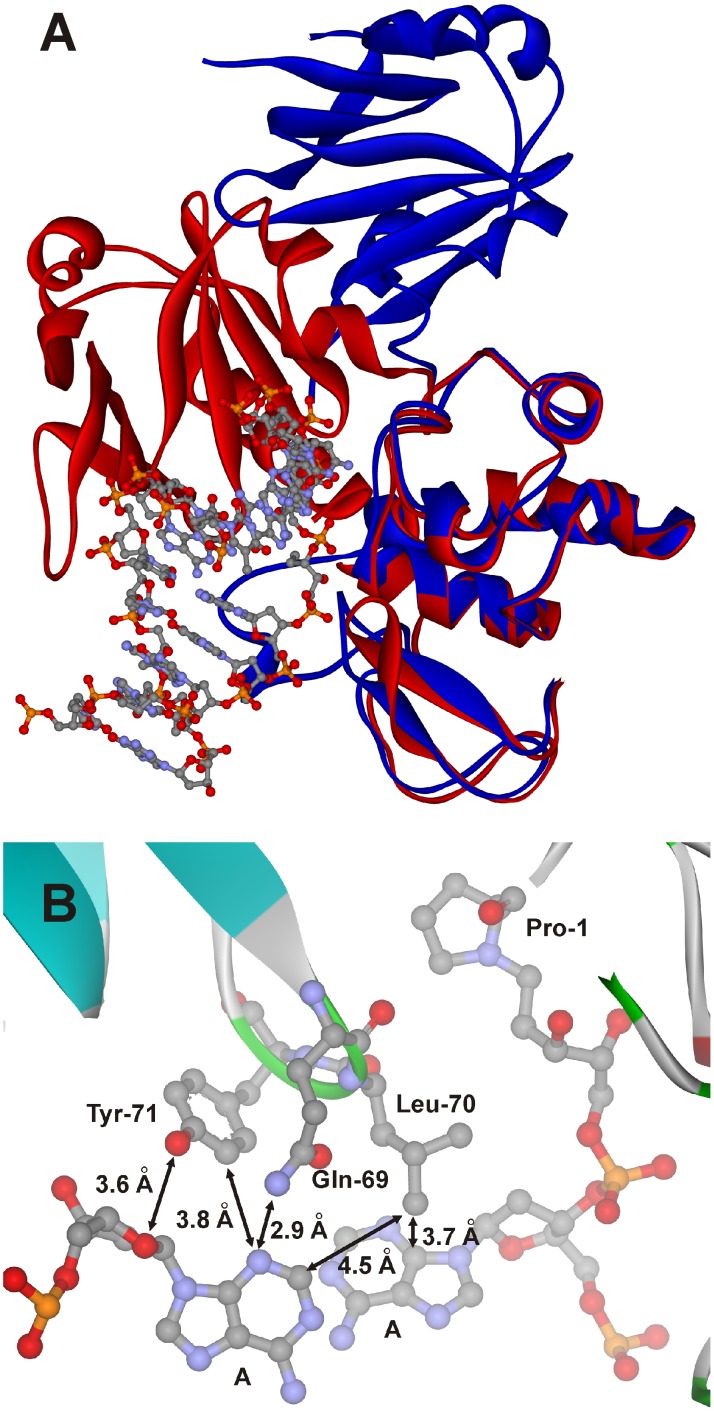
Overall structure of the Nei and close-up view of the active site. (A) The structures of the free Nei (blue, PDB ID 1Q39) and Nei covalently complexed with damaged DNA (red, PDB ID 1K3W). (B) The Gln-69, Leu-70, and Tyr-71 triad inserted into the duplex. The arrows indicate the distances between Tyr-71 Cε2 or Gln-69 Nε2 and *N*3 of the A base placed opposite of the damaged nucleotide (3.8 Å or 2.9 Å, respectively); between Tyr-71 Oη and O4' of the nucleotide (3.6 Å); between Leu-70 Cδ1 and C2 of the A base placed opposite of the damaged nucleotide (4,5 Å) or C4 of the A base placed on the 5′-side of the damaged nucleotide (3.7 Å).

The overall structures of the free Nei and Nei covalently complexed with damaged DNA are depicted in Figure 5A [30], [31]. As shown in Figure 5B, base placed on the 5′-side of the damaged nucleotide has the ability to interact only with Leu-70 (shortest distance is 3.7 Å). At the same time, the base placed opposite of damaged nucleotide interacts with Gln-69 (2.9 Å) and Tyr-71 (3.8 Å). Therefore, the bases placed opposite and on the 5′-side of the damaged nucleotide were selected for substitution by the fluorescent base analogues. The canonical and labeled ODNs used in this study are presented in Table 1. They contain a dihydrouracyl base (DHU), as a substrate for Nei,

**Table 1:** ODN sequences used in this work.

Shorthand	Sequence
**DHU/X**	5**′**-CTCTC**(DHU)**CCTTCC-3**′**
**X** = G, aPu, C^py^, tC^O^, 3HC	3**′**-GAGAG **X** GGAAGG-5**′**
**Y-DHU**	5**′**-CTCT**Y**(DHU)CCTTCC-3**′**
**Y** = aPu, 3HC; **Z** = C	3**′**-GAGA**Z** G GGAAGG-5**′**
**Y** = C^py^; **Z** = G	

Since the DNA is known to undergo conformational transitions upon binding by Nei, the stopped-flow kinetic assay was used to measure the changes of fluorescence intensities of the various DNA base labels in the model DNA-substrates.

## Materials and Methods

### Oligonucleotides and Enzymes

Nei protein was purified as described [31]. The concentration of the active enzyme (∼80%) was determined by trapping with NaBH_4_ as described [34] using the ODN duplex containing a DHU/G pair; the reported concentrations of Nei are those of the active form. The ODNs (Table 1) were synthesized by established phosphoramidite methods on an ASM-700 synthesizer (BIOSSET Ltd., Novosibirsk, Russia) from phosphoramidites purchased from Glen Research (Sterling, VA) in the Laboratory of Bionanotechnology of ICBFM. 3HC phosphoramidite was synthetized as described previously [22], [23]. Synthetic oligonucleotides were unloaded from the solid support with ammonium hydroxide according to manufacturer’s protocols. Deprotected oligonucleotides were purified by HPLC. The purity of ODNs exceeded 98% as estimated by electrophoresis in 20% denaturing PAGE after staining with the Stains-All dye (Sigma-Aldrich). Concentrations of oligonucleotides were determined from their absorbance at 260 nm. ODN duplexes were prepared by annealing modified and complementary strands at a 1∶1 molar ratio.

### Stopped-flow Fluorescence Measurements

Stopped-flow measurements with fluorescence detection were essentially carried out as described [35], [36]. A model SX.18MV stopped-flow spectrometer (Applied Photophysics Ltd, Leatherhead, UK) fitted with a 150 W Xe arc lamp and 2 mm path length optical cell was used. The dead time of the instrument was 1.4 ms. Experiments were performed at 10°C in the buffer containing 50 mM Tris-HCl (pH 7.5), 50 mM KCl, 9% (v/v) glycerol, 1 mM DTT, 1 to 3 µM Nei and 1 µM DNA substrates. The excitation wavelengths were 290, 310, 344, 360 and 375 nm for the Trp, aPu, C^py^, tC^O^ and 3HC fluorescent dyes, respectively. The emission was monitored using a 320 nm long pass wavelength filter for Trp, 370 nm long pass wavelength filter for aPu and C^py^, 395 nm long pass wavelength filter for tC^O^ and 3HC. Additionally, to separate detection of the second emission T* band of 3HC fluorophore, 495 nm long pass filter was used. Typically, each trace shown is the average of four or more traces obtained in individual experiments. Data obtained from the stopped-flow kinetic assays and pre-steady-state kinetic parameters for possible kinetic schemes were determined as described previously [34].

To calculate the intensity ratio (*I*_N_*/*I*_T_*) for each time point, the equation (1) was used.
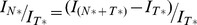
(1)where *I*_(N*+T*)_ is the total N*+T* fluorescence intensity (395 nm filter), *I*_T_* is the T* fluorescence intensity (495 nm filter) at the same time point of the kinetic curves.

### PAGE Time-course Experiments

The reaction mixture included 2 µM Nei, 2 µM ODN substrate, 50 mM Tris-HCl (pH 7.5), 50 mM KCl, 9% (v/v) glycerol, and 1 mM DTT. All experiments were conducted at 10°C. The modified strands of the ODN substrates were 5′-end labeled using T4 polynucleotide kinase (New England Biolabs, Beverly, MA) and γ[32P]-ATP (4500 Ci/mol) (Radioizotop, Moscow, Russia) according to the manufacturer’s protocol. The reactions were initiated by adding the enzyme. Aliquots (2 µL) of the reaction mixture were quenched with 3 µL of gel-loading dye containing 7 M urea, and loaded on a 20% (w/v) polyacrylamide/7 M urea gel. The disappearance of substrate and the formation of product were analyzed by autoradiography and quantified by scanning densitometry using Gel-Pro Analyzer v4.0 software (Media Cybernetics, Bethesda, MD).

### Molecular Modeling

As a complement to experimental studies, we performed molecular dynamics (MD) simulations using the BIOPASED program [37] with implicit Gaussian Shell solvation model extended for DNA [38]. Structures of eight DNA duplexes have been modeled by MS simulated annealing at 300 K. Comparison of conformational distortion of duplexes structures due to fluorescent nucleotide analog are performed at 50 K to elucidate structural differences between average structures. 3HC analog has the largest structural distortion from the ideal B-DNA ∼1.2 Å and largest amplitude of thermal fluctuations at 300 K comparing to all other analogs investigated.

## Results and Discussion

**Figure 6:**
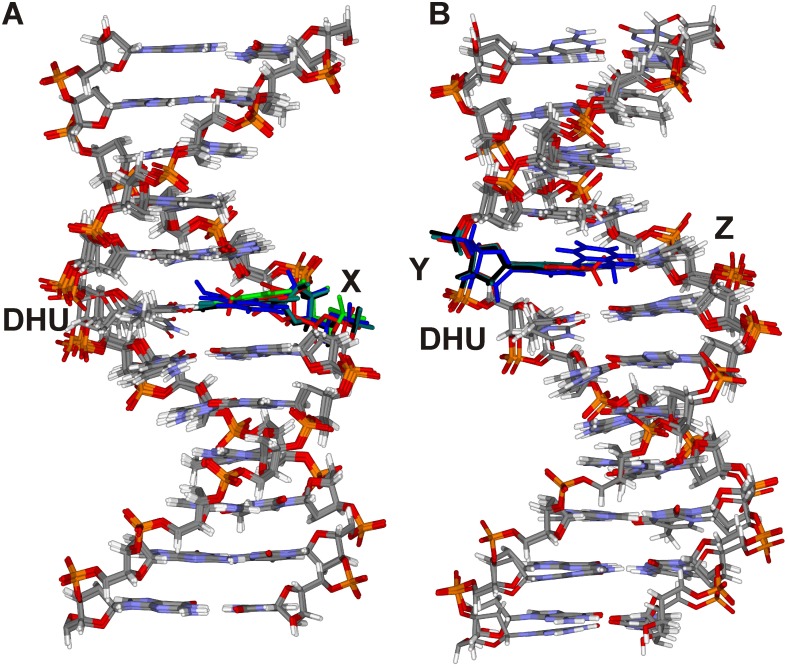
Comparison of the MD simulations of DNA duplexes. The MD simulation of DHU/X (A) and Y-DHU (B) model duplexes. The colors correspond to the duplexes containing aPu (black), C^py^ (red), tC^O^ (green), 3HC (blue), unlabeled duplex (dark green).

First, molecular dynamics (MD) simulation of the structures of all used DHU/X and Y-DHU model duplexes was performed to verify that the labels have minimal impact on the duplex structure (Fig. 6). It was shown that fluorescent labels did not disturb noticeably the regular structure of DNA double helix. The maximum deviation of DHU base in these duplexes is 1.2 Å, i.e. in the range of thermal fluctuation of the canonical DHU/G duplex.

**Figure 7:**
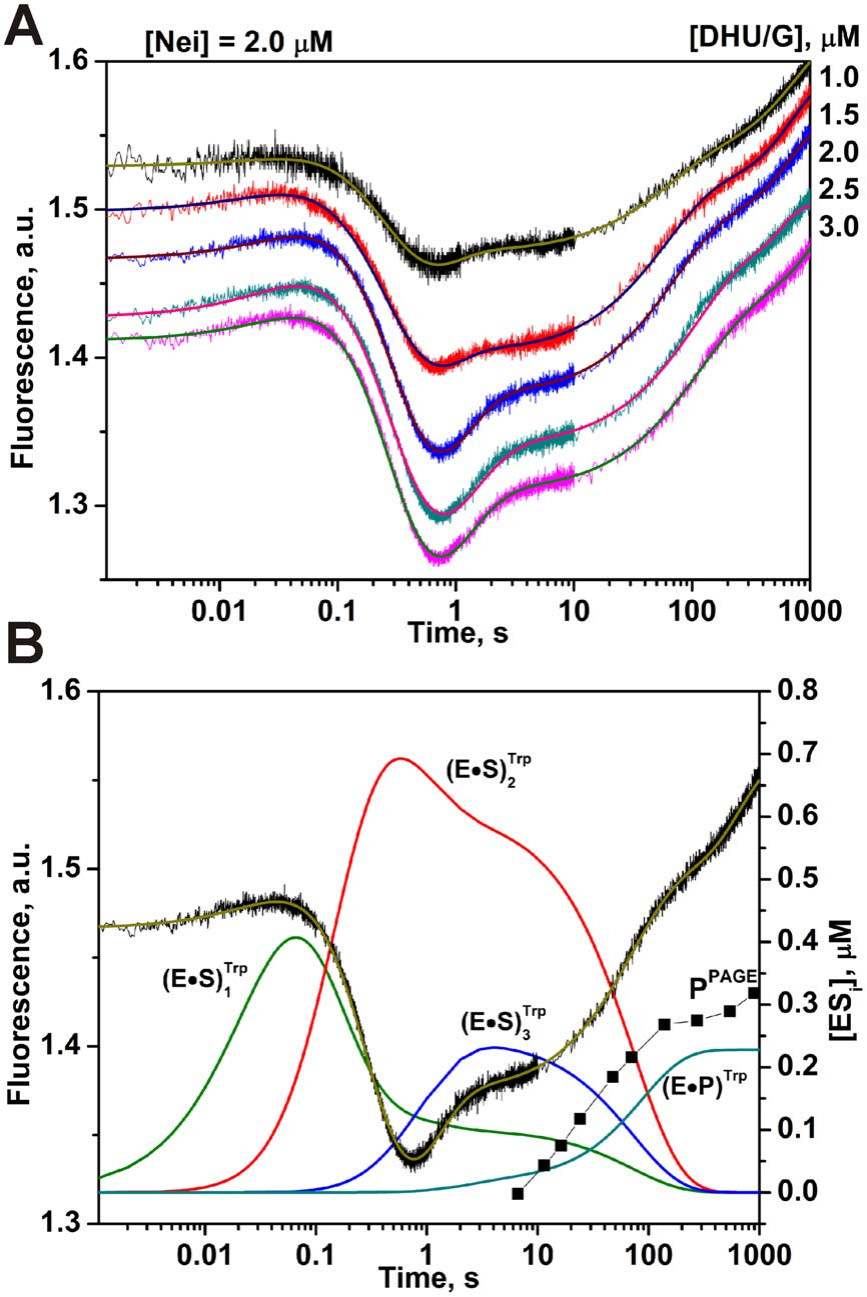
Interaction of Nei with DHU/G-substrate. (A) Changes in Trp fluorescence intensity during the interaction of Nei with DHU/G-substrate. Jagged traces display the experimental data whereas smooth curves correspond to the fit of the data to the kinetic model (Fig. 3). Concentrations of the DNA (µM) are shown next to the plots. (B) Time-course of appearance and disappearance of transient enzyme–substrate complexes during the cleavage of DHU/G by Nei. Modeling was done for 2 µM Nei and 2 µM substrate with the rate constants listed in Table 2. Accumulation of the reaction products detected by PAGE analysis (P^PAAG^) is shown for comparison.

Secondly, the duplex DHU/G was used as a control of the enzyme activity. The structural data reveal gross movements in the structure of Nei upon DNA binding, with the *N*-terminal and *C*-terminal domains “closing” by ∼50° to assemble the enzyme’s active site (Fig. 5) [30], [31]. The conformational changes of the enzyme during DNA-substrate binding, lesion recognition and excision were registered by the intrinsic fluorescence of the enzyme’s Trp residues [34]. As depicted in Figure 7A, complex fluorescence dynamics was observed as it was expected based on previous data [34]. The minimal kinetic scheme describing the observed changes of Trp fluorescence intensity was identical to scheme in Figure 3 and contained three equilibrium steps that characterized substrate binding followed by an irreversible chemical step and then an equilibrium step of product release. The rate constants of the elementary steps estimated according to this kinetic scheme are listed in Table 2. It should be noticed that formation of the first enzyme/substrate complex (E•S)_1_^Trp^ proceeded during the first 50 ms. The maximum of the concentration of the second complex (E•S)_2_^Trp^ was achieved at 0.6 s, whereas catalytically active complex (E•S)_3_^Trp^ was formed at 5–10 s. The irreversible base excision and strand cleavage, as well as the product release are undistinguishable following Trp fluorescence and proceeded at times >20 s. The formation of E•P complex in the chemical step registered by Trp fluorescence changes is in good agreement with the accumulation of the reaction products detected by PAGE analysis (Fig. 7B). The first reversible step likely corresponds to the initial binding, which changes the shielding of the Nei Trp residues. The two remaining steps discernible by Trp fluorescence may reflect the insertion of amino acid triad into DNA, in exchange of the damaged base everted into the enzyme’s active site, as well as movement of the zinc finger resulting in the fine-tuning of the enzyme active site. These data are consistent with the hypothesis that Nei maintains a stable conformation during the breakage of the covalent bonds in DNA (Fig. 3) [34]. It is noteworthy that the *N*-glycosidic bond cleavage is faster than the *β*-elimination process, making of this latter the rate-limiting step.

**Table 2:** The rate constants for interactions of Nei with DHU/G and DHU/X-substrates.

Constants	Trp	3HC	tC^O^	C^py^
*k*_1_, M^−1^s^−1^	(10.5±6.2)×10^6^	(53±10)×10^6^	(200±70)×10^6^	(115±30)×10^6^
*k_-_*_1_, s^−1^	6.1±3.4	69±22	380±150	246±44
*k*_2_, s^−1^	9.5±2.4	3.9±1.7	0.46±0.16	0.52±0.11
*k_-_*_2_, s^−1^	1.6±0.2	4.8±1.5	0.005±0.002	2.5±0.2
*k*_3_, s^−1^	0.4±0.1	0.30±0.08	-	-
*k_-_*_3_, s^−1^	0.9±0.2	0.4±0.1	-	-
*k*_cat_, s^−1^	0.056±0.027	0.014±0.003	0.035±0.015	0.040±0.028
*K*_P_, M	(6.5±3.4)×10^−6^	(0.16±0.08)×10^−6^	(6.0±2.5)×10^−6^	(3.6±2.1)×10^−6^

**Figure 8:**
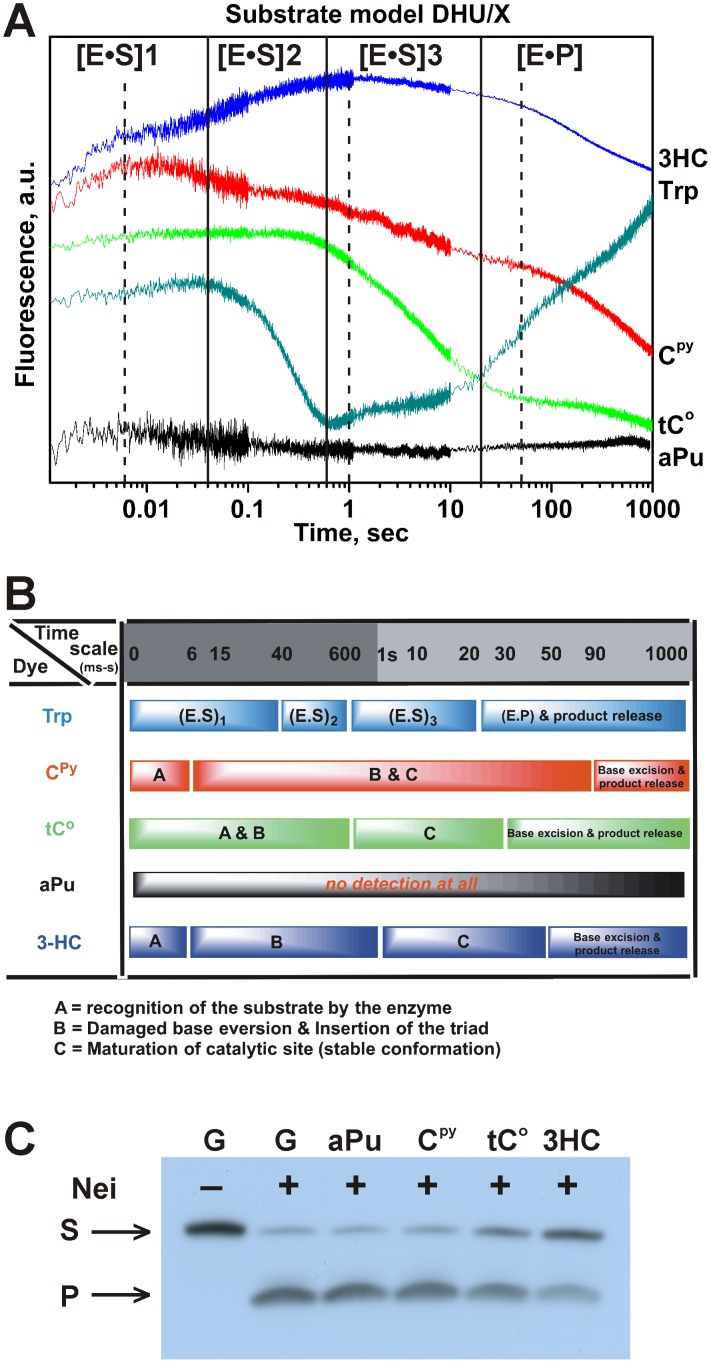
Interaction of Nei with DHU/X-substrates. (A) Changes in Trp, aPu, C^py^, tC^O^ and 3HC fluorescence intensity during the interaction of 2 µM Nei and 1 µM DNA substrates, containing fluorescent nucleotides opposite the damaged nucleotide. The emission of the long-wavelength T* band (495 long wavelength pass filter) of 3HC dye is presented. The black vertical lines delimit the 4 kinetic steps of protein dynamics following Trp fluorescence. The dashed black vertical lines delimit the 4 kinetic steps of DNA dynamics following 3HC fluorescence. (B) Summary of the different steps detected by the different fluorescent reporters used in this study. (C) Effect of the fluorescent labels on the enzymatic activity of Nei. The concentrations of Nei and DNA substrates were 2 µM and 1 µM, respectively.

We then analyzed the DNA dynamics following the fluorescence intensity of the label (C^py^, aPu, tC^O^ or 3HC) in time. Previously, using C^py^ opposite to the damaged base, significant changes of C^py^ fluorescent intensity were registered during interaction of the Fpg (the structural homolog of Nei) with DNA [17]. According to the analogy with Fpg, we tried to register the DNA conformational changes in the course of interaction with Nei using the DHU/X model duplexes containing fluorescent labels (C^py^, aPu, tC^O^ or 3HC) opposite to the damaged base (Fig. 8A&B). It is important to notice that the processes of structural rearrangements in protein and DNA can occur with a time shift relative to each other, because of an intrinsic inertia of the DNA conformational and environmental changes induced by protein interactions (Fig. 8A&B). As shown in Figure 8C, aPu and C^py^ did not affect the Nei activity and both tC^O^ and 3HC fluorescent base mimics retained substantial enzymatic activity [39].

**Figure 9:**
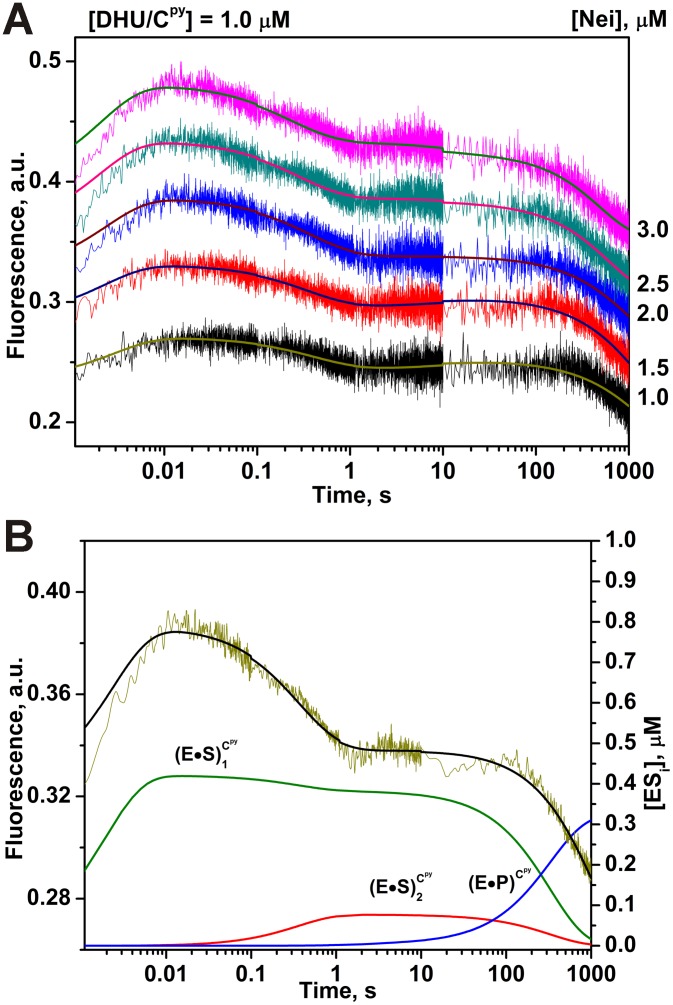
Interaction of Nei with DHU/C^py^-substrate. (A) Changes in C^py^ fluorescence intensity during the interaction of Nei with DHU/C^py^-substrate. Jagged traces show the experimental data, smooth curves correspond to the fit of the data to the kinetic model (Fig. 10). Concentrations of the DNA are shown next to the plots. (B) Time-course of appearance and disappearance of transient enzyme–substrate complexes during the cleavage of DHU/C^py^ by Nei. Modeling was done for 2 µM Nei and 1 µM substrate with the rate constants listed in Table 2.

**Figure 10:**
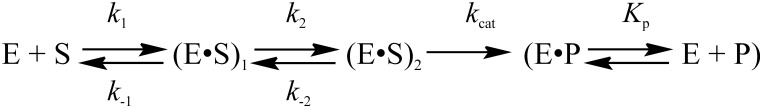
Kinetic mechanism of Nei processing of the DHU/C^py^- and DHU/tC^O^-substrates.

The aPu fluorescence did not change at all (Fig. 8A&B). The absence of the aPu signal change denotes that the most widely used fluorescent base is unable in this example to sense the DNA conformational transitions occurring during protein-DNA interactions when the dye is placed opposite of the damaged base. As shown in Figures 9A&B, fluorescence of C^py^ exhibited three different steps. On the basis of data (Fig. 9A), the minimal kinetic scheme (Fig. 10) was proposed for the interaction between Nei and DHU/C^py^-substrate whereas the values for rate constants were reported in Table 2. Figure 9B illustrates the time-course of appearance and disappearance of the different intermediates as evidenced from C^py^ fluorescence. The fast increase during 10 ms likely corresponds to the beginning of the first stage detected by Trp fluorescence. The gradual decrease of C^py^ fluorescence between 10 ms and 10 s can be attributed to the end of the first stage and to the formation of complexes (E•S)_2_^Trp^ and (E•S)_3_^Trp^, which cannot be distinguished by C^py^ fluorescence. The significant decrease of C^py^ intensity after 90 s is likely connected to complex (E•P)^Trp^ and product release.

**Figure 11:**
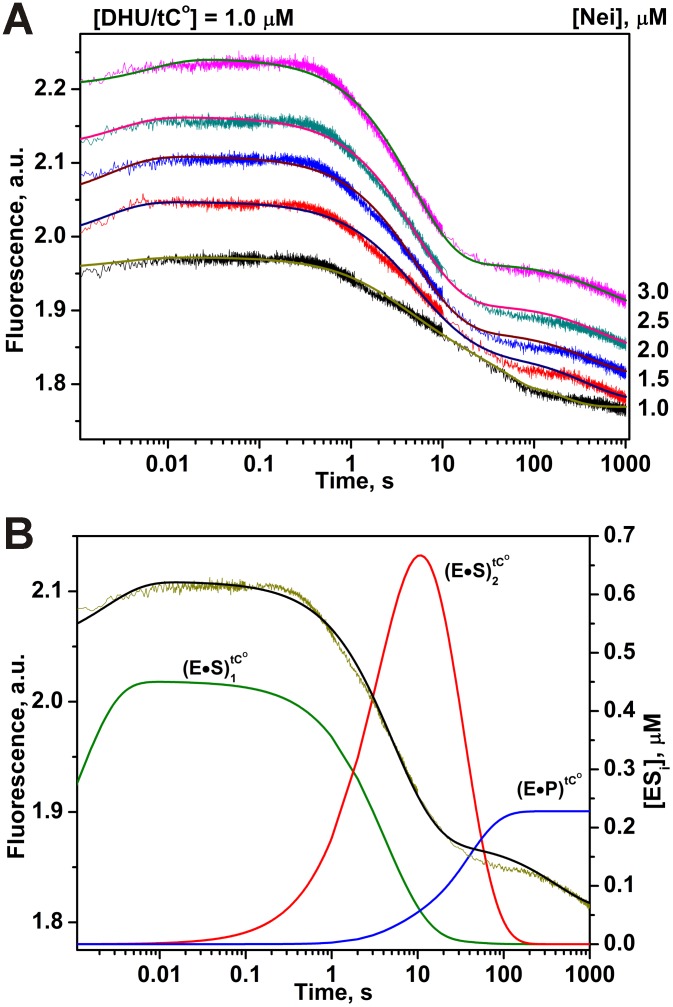
Interaction of Nei with DHU/tC^O^-substrate. (A) Changes in tC^O^ fluorescence intensity during the interaction of Nei with DHU/tC^O^-substrate. Jagged traces show the experimental data, smooth curves correspond to the fit of the data to the kinetic model (Fig. 10). Concentrations of the DNA are shown next to the plots. (B) Time-course of appearance and disappearance of transient enzyme–substrate complexes during the cleavage of DHU/tC^O^ by Nei. Modeling was done for 2 µM Nei and 1 µM substrate with the rate constants listed in Table 2.

Using the tC^O^ dye, it was also possible to register the processes of three different DNA conformational transitions (Fig. 11A&B) during interaction with Nei but different from C^py^ (Fig. 9A&B). The initial slight increase of the tC^O^ fluorescence during 0.6 s characterizes the formation of (E•S)_1_^Trp^ and (E•S)_2_^Trp^ complexes in correspondence with Trp fluorescence data. The decreasing phase of the fluorescence intensity after 1 s up to 30 s is reasonably attributed to the (E•S)_3_^Trp^ complex. The catalytic steps and dissociation of the complex of enzyme with the reaction product (E•P)^Trp^ marginally influence on the tC^O^ fluorescence. Only two binding equilibria were sufficient to describe conformational changes in DNA in minimal terms (Fig. 10). Table 2 lists the values of rate constants determined for this scheme.

**Figure 12:**
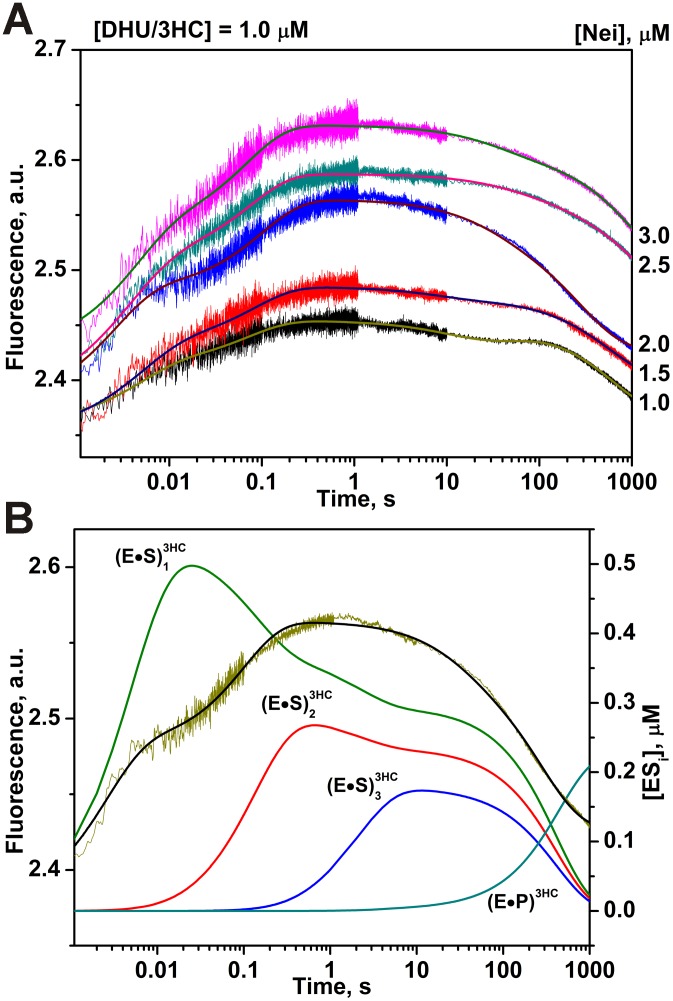
Interaction of Nei with DHU/3HC-substrate. (A) Changes in 3HC fluorescence intensity during the interaction of Nei with DHU/3HC-substrate. Jagged traces show the experimental data, smooth curves correspond to the fit of the data to the kinetic model (Fig. 3). Concentrations of the DNA are shown next to the plots. (B) Time-course of appearance and disappearance of transient enzyme–substrate complexes during the cleavage of DHU/3HC by Nei. Modeling was done for 2 µM Nei and 1 µM substrate with the rate constants listed in Table 2.

By contrast to the C^py^ and tC^O^ labels, the kinetic curves obtained using 3HC label showed four steps (Fig. 12A&B) as were found by measurements of Trp fluorescence (Fig. 3). As for C^py^ label, it demonstrates the fast initial increase of the fluorescence intensity, which is connected with the early formation of (E•S)_1_^3HC^ complex. However, it is also able to differentiate the formation of a second DNA conformational change (complex (E•S)_2_^3HC^) as evidenced by the second intensity increase phase up to 1 s. The second phase is likely related to the end of the formation of (E•S)_1_^Trp^ and to (E•S)_2_^Trp^. Between 1 and 50 s, the third step is accompanied by a small decrease in the fluorescence signal and leads to the formation of catalytically active complex (E•S)_3_^3HC^. The dissociation of enzyme-product complex (E•P)^3HC^ returns the fluorescence intensity to the initial level (Fig. 12A). Therefore, the difference in Trp and 3HC fluorescence changes during interactions is more informative compared to the other dyes, that allows correlating more precisely the fluorescence variations to the conformational changes of enzyme and DNA.

Comparison of the individual rate constants (Table 2) for processes detected by different fluorophores (Trp, C^py^, tC^O^ and 3HC) has shown that DNA faster responds to the initial binding. The rate constant *k*_1_, which characterizes DNA conformational change, is at least 5 times larger than the same rate constant for enzyme conformational change. Probably, this is due to the fact that the change in intensity of Trp residues occurs only with significant conformational changes of the protein, such as the domains “closing”. The rate constants *k*_2_ of the second binding step detected by Trp and 3HC differ insignificantly (in 2.4 times), suggesting that both fluorophores characterize the sequentially coordinated processes, which can be DNA bending, DHU base eversion and insertion of the amino acids of enzyme into the DNA duplex. Interesting to note that rate constants *k*_3_ of the third binding step detected by Trp and 3HC and rate constants *k*_2_ of the second binding step detected by C^py^ and tC^O^ have close values, indicating that rearrangements of the active site to achieve the catalytic structure requires mutual simultaneous conformational changes of enzyme and DNA. The rate constants *k*_cat_ of the catalytic step are a little reduced (∼1.5 times) for DHU/C^py^- and DHU/tC^O^-substrates in comparison with DHU/G-substrate. For DHU/3HC-substrate this value differs from DHU/G-substrate by a factor 4 that is in accordance with the qualitative data obtained for PAGE analysis of the reaction product (Fig. 8C).

**Figure 13:**
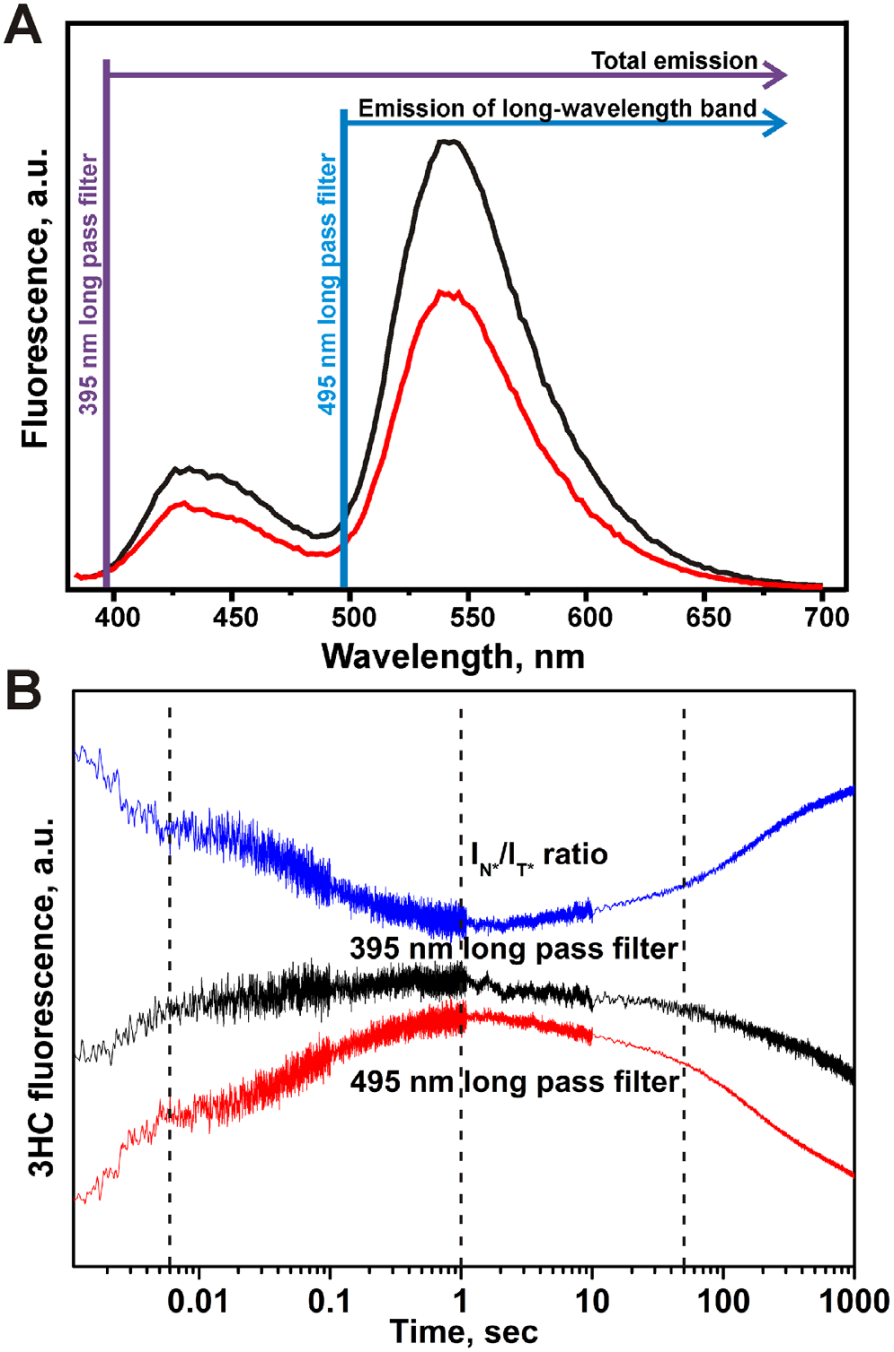
Fluorescent properties of 3HC dye. (A) Steady-state emission spectra of 3HC (2 µM) in 3HC-DHU single- (black) and 3HC-DHU/GGC double-stranded (red) states. (B) Intensity changes of total (N*+T*) and long-wavelength T* emissions of 3HC and N*/T* ratio during the interaction of Nei with DHU/3HC substrate. The concentrations of Nei and DNA substrates were 2 µM and 1 µM, respectively. The dashed black vertical lines delimit the 4 kinetic steps of DNA dynamics following 3HC fluorescence intensity ratio.

To get further insight about the mechanism of Nei interacting with DNA, we examined in time the intensity ratio changes of the dual emissive 3HC labels. Single and double stranded labeled ODNs displayed the two well-resolved emission bands of 3HC, with the short- and long-wavelength maxima (N* and T*) centered at 430 nm and 540 nm, respectively (Fig. 13A). For both single and double stranded ODNs, the intensity ratio of the two bands (*I*_N_*/*I*_T_*) was close to 0.3. These results are consistent with the previously reported data and indicate that the environment of the labeling site is mainly aprotic and have medium polarity [23]. The different long pass filters for the 3HC dye were tested in order to distinguish the changes of the total emission corresponding to both short- and long-wavelength bands (N* & T* -395 nm long pass filter) from the only emission of the long-wavelength band (495 nm long pass filter) (Fig. 13B).

As described above four discernible stages were observed on kinetic curves using 3HC (Fig. 12A&B). Moreover, the amplitudes and the slopes of fluorescence intensity changes for the various stages are different for both channels indicating a change of the intensity ratio of the two emissive bands in time (Fig. 13B). Since the total N*+T* (395 nm long pass filter) and the T* (495 nm long pass filter) emissions were collected separately, the kinetic curves of the intensity ratio *I*_N_*/*I*_T_* were calculated.

The first stage of the DHU/3HC duplex binding led to a decrease of the intensity ratio up to 6 ms (Fig. 13B). This stage could be associated with the fast formation of the encountered nonspecific Nei•DNA complex because of binding to protein should reduce DNA structural fluctuations and increased shielding from water molecules. This interpretation is supported by the fact that the hydration and hydrogen bond network of DNA are dramatically affected by the protein interactions [40]. Then, intensity ratio I_N*_/I_T*_ did not change in the time interval between 6 ms and 20 ms. Afterwards, a second decrease occurred up to 1.0 s indicating that the microenvironment of the dye is further shielded from water molecules and exhibited weaker dipole/dipole interactions. The decreasing phase of *I*_N_*/*I*_T_* ratio can be associated with the DNA bending, DHU base eversion and subsequent amino acids insertion, that is in the agreement with Trp data. The *I*_N_*/*I*_T_* ratio exhibited only marginal 3HC fluorescence change between 1 and 50 s. This stage should correspond to the formation of the productive complex. The last stage for the cleavable DHU/3HC substrate showed increase of intensity ratio *I*_N_*/*I*_T_* up to the initial value indicating the returning of the microenvironment of 3HC of the DNA that is more water exposed (Fig. 13B). The last step is therefore compatible with the dissociation of the enzyme-product complex and the release of the DNA product.

**Figure 14:**
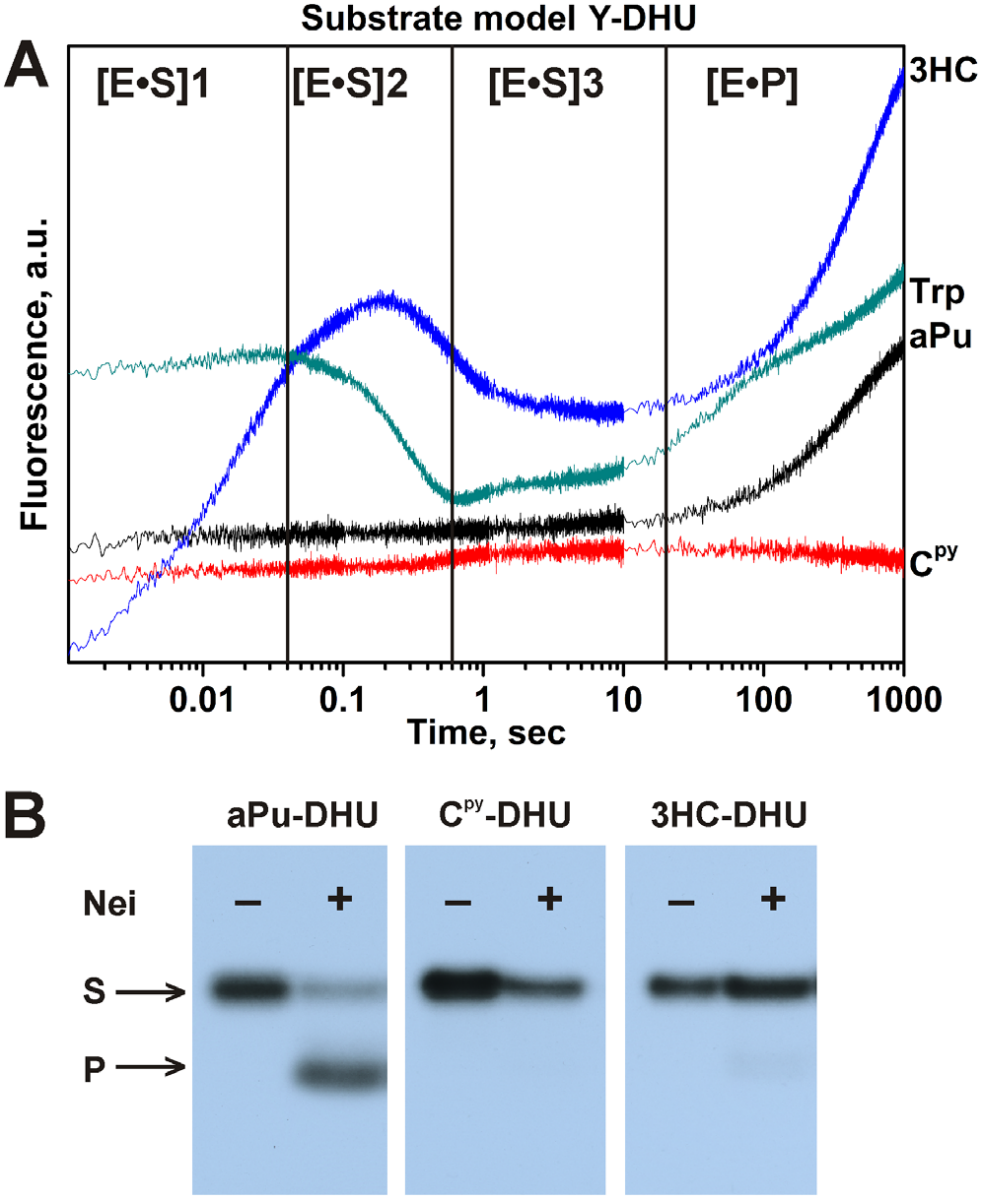
Interaction of Nei with Y-DHU-substrates. The changes in Trp, aPu, C^py^ and 3HC fluorescence intensity during the interaction of Nei with DNA substrates, containing fluorescent nucleotides on the 5**′**-side of the damaged nucleotide. The emission of the long-wavelength T* band (495****nm long pass filter) of 3HC dye is presented. The black vertical lines delimit the 4 kinetic steps of protein dynamics following Trp fluorescence. (B) The effect of the fluorescent labels on the enzymatic activity of Nei. The concentrations of Nei and DNA substrates were 2** µ**M and 1** µ**M, respectively.

To develop the comparison of the fluorophores the model Y-DHU duplexes with different location of the fluorescent labels were used (Table 1). In this model system, the fluorescent dye (aPu, C^py^, 3HC) is placed between T and damaged base DHU, which should be flipped out and removed upon the interaction with Nei. The DNA dynamics following in time the fluorescence intensity of the label was monitored (Fig. 14A).

**Figure 15:**
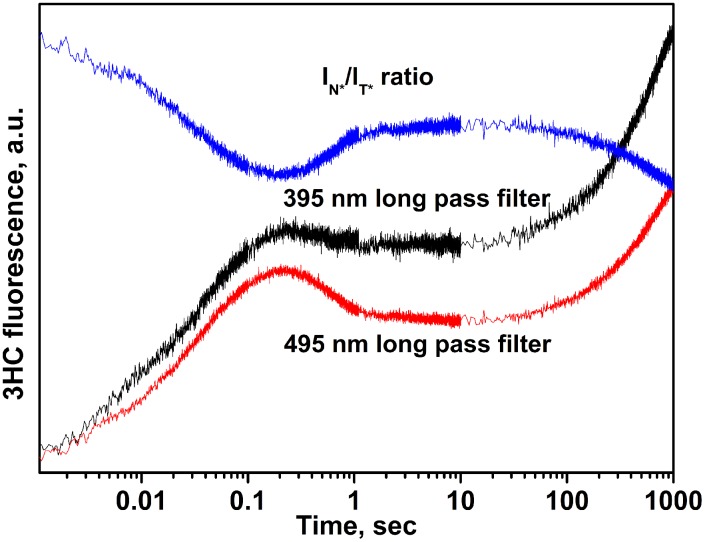
Stopped-flow fluorescence traces for interactions of Nei with 3HC-DHU-substrate. Intensity changes of total (N*+T*) and long-wavelength T* emissions of 3HC and N*/T* ratio during the interaction of Nei with 3HC-DHU-substrate. The concentrations of Nei and DNA substrate were 2 µM and 1 µM, respectively.

The interaction of Nei with aPu-DHU substrate presented no fluorescence changes up to 100 s despite the fact that the enzyme cleaves this substrate with good efficiency (Fig. 14B). Again aPu fluorescent label is not sensitive for binding by Nei whereas in our previous works related with hOGG1 and Fpg enzymes this fluorescent base was a very informative probe for the study of enzyme-DNA interactions [12]–[14], [41], [42]. The increase in the aPu fluorescence intensity at times >100 s can be the result of the transition to the states with poorer stacking and/or quenching abilities of the neighbor bases as a consequence of the strand cleavage and dissociation of the enzyme-product complex (Fig. 14). The binding of Nei with C^py^-DHU substrate displayed the slight increase of the C^py^ fluorescence up to 10 s (Fig. 14). In contrast to the DNA substrates containing aPu and C^py^, the duplex 3HC-DHU containing the 3HC fluorophore showed at least four discernible stages as observed with the DHU/3HC model (Fig. 14&15). However, the PAGE analysis of the product accumulation showed that C^py^ and 3HC labels deprive the enzyme of its catalytic activity (Fig. 14B) by contrast to aPu. In the case of C^py^ or 3HC, the introduction of the sterically more demanding labels on the 5′-side of DHU in the DNA duplex may inhibit the formation of the catalytically competent state of enzyme (*vide infra*).

In the case of 3HC-DHU duplex, the fast change of the intensity ratio again occurs up to 10 ms. It was clearly discernable when analyzing the intensity ratio channel indicating about formation of the nonspecific Nei•DNA complex (compare Fig. 14&15). A second phase takes place in the time interval between 0.01 s and 0.2 s. It can be suggested that this stage corresponds to the insertion of the amino acids into DNA in exchange of the everted damaged base. The next stage was reached at 1 s, indicating an additional change of the dye environment. Between 1 and 70 s, the next step is accompanied by a marginal change in the fluorescence signal. The last phase was observed above 70 s by a net change of the fluorescence signal (Fig. 14&15). Kinetic curves for uncleavable 3HC-DHU-substrate was evidenced by a decrease in the *I*_N_*/*I*_T_* ratio up to 1000 s (Fig. 15) indicating the attempts of the enzyme to further disturbance of the duplex structure. Indeed, for a productive enzyme-substrate complex, an increase of the *I*_N_*/*I*_T_* ratio should be observed as a consequence of the dye more exposed to water in the final product (CTCT3HC). Therefore the decrease in the *I*_N_*/*I*_T_* ratio for 3HC-DHU is indicative of the formation of a non-productive complex as supported by PAGE analysis of the product accumulation (Figures 14B&15).

## Conclusions

To sum up, monitoring of the Trp and 3HC fluorescence (Figures 7A&B, 12A&B and 13B) is complementary and allows following the protein and DNA dynamics, respectively. It gives a detailed picture of the mutual sequential conformational adjustment of enzyme and DNA substrate up to the formation of an active complex and release of the product. The initial Trp fluorescence growth, which is expressed in an increase of the 3HC fluorescence intensity up to 6 ms for DHU/3HC, probably characterizes the fast process of the enzyme domains “closing” and formation of the first nonspecific complex. The DNA conformation and environment changes are reflected by the increase of the quantum yield of 3HC label, probably due to reduced neighboring G, C and water-mediated quenching and reduced DNA structural fluctuations (*16*). On the other hand, they are characterized by a decrease of the intensity ratio *I*_N_*/*I*_T_*, indicating that the dye is more shielded from water molecules and that dipole/dipole interactions are weaker (Figure 13B). The analysis of fluorescence curves showed that the first change of the Trp fluorescence intensity (up to 0.6 s) consists of two 3HC fluorescently discernible conformational changes in DNA (the plateau between 0.006 s and 0.02 s and an intensity increase up to 1.0 s). Probably, these phases of 3HC fluorescence are corresponding to the DNA bending, DHU base eversion and subsequent insertion of the amino acid triad Gln-69, Leu-70, and Tyr-71 into the DNA duplex. These processes lead to increase in hydrophobicity of the medium near the 3HC resulting in the decrease of the 3HC fluorescence intensity ratio. The next growth of the Trp fluorescence signal (in the time interval from 1 s to 10 s) results in a marginal change of the 3HC fluorescence (DHU/3HC-substrate). In this time range, the catalytic complex should be formed as the consequence of the fine-tuning of the Nei active site structure. The last change in the Trp and 3HC fluorescence (DHU/3HC-substrate) intensities at times >10 s can be the result of the formation and the dissociation of the enzyme-product complex exposing the dye to a more hydrated environment of the DNA product. The increase of the *I*_N_*/*I*_T_* ratio of the 3HC label supports this idea. On the other hand, the 3HC-DHU duplex exhibited also at least four discernible stages but finished to a non-productive enzyme-substrate complex as supported when analyzing the intensity ratio channel and the product accumulation on PAGE. Non-productive enzyme-substrate complex was also observed with the C^py^-DHU substrate indicating that position 5′ to DHU is sensitive to substitution with labels and critical to catalysis.

Thereby, comparison of the one-band emissive fluorescent nucleobase analogues (aPu, C^py^ and tC^O^) with the new dual emission label 3HC showed that, the usage of the multichannel 3HC label gave us the possibility to specify more exactly the nature of steps registered previously by Trp fluorescence changes (Fig. 3) [34]. Therefore, it was possible to propose a new kinetic mechanism which includes three reversible steps followed by an irreversible step and DNA product release, corresponding to: (1) the fast initial DNA binding and formation of nonspecific Nei•DNA complex, (2) DNA bending at lesion site, damaged base eversion from the duplex and insertion of amino acids of the enzyme (Gln-69, Leu-70, and Tyr-71) into the DNA void, (3) adjustment of the active site to catalytically competent state, (4) the hydrolysis of the *N*-glycosidic bond, the elimination of the 3′-phosphate (*β*-elimination) followed by the release of the 5′-phosphate (*δ*-elimination), and the last stage (5) that fits with dissociation of the complex between DNA-product and enzyme.
